# Case Report: From standard therapy refusal to rare metastases: a case of untreated HER2-positive breast cancer progressing to brain and thyroid metastases after seven years of follow-up during which the patient received only Chinese herbal medicine

**DOI:** 10.3389/fonc.2026.1861088

**Published:** 2026-07-22

**Authors:** Qing Yu, Jian Zhang, Hui Li, Kang He

**Affiliations:** 1 Department of Pathology, Deyang People’s Hospital, Deyang, China; 2 Department of Ultrasound, Deyang People’s Hospital, Deyang, China

**Keywords:** brain metastasis, case report, Chinese herbal medicine, HER2-positive breast cancer, thyroid metastasis, treatment refusal

## Abstract

**Objective:**

HER2-positive breast cancer is an aggressive subtype associated with high rates of early recurrence and brain metastasis. Standard adjuvant therapy, including anti-HER2 targeted agents, significantly improves outcomes. This case illustrates the natural history of untreated HER2-positive breast cancer and documents an exceptionally rare metastatic site.

**Methods:**

We report a case of a 63-year-old woman with HER2-positive (3+, FISH amplified), ER-positive (80-90%), PR-negative, Ki-67 15% invasive ductal carcinoma of the left breast (pT1cN2aM0, 7/12 axillary lymph nodes positive).

**Results:**

The patient refused all standard adjuvant therapies (chemotherapy, radiotherapy, endocrine therapy, and anti-HER2 therapy) and opted for sole Chinese herbal medicine. After seven years of follow-up (with a disease-free interval of approximately four years, she developed a right cerebellar hemisphere metastasis (2.3×2.7 cm with extensive edema), widespread lymphadenopathy, and a pathologically confirmed thyroid metastasis—an extremely rare event in breast cancer. Fine-needle aspiration of the thyroid nodule demonstrated atypical cells, and subsequent immunohistochemistry confirmed breast cancer origin (GATA3+, GCDFP-15+, HER2 3+, ER+, TTF-1-, TG-).

**Conclusions:**

This case provides compelling *in vivo* evidence of the aggressive natural history of untreated HER2-positive breast cancer. The development of brain metastasis underscores the critical importance of anti-HER2 agents that penetrate the blood-brain barrier. The rare thyroid metastasis adds to the literature on unusual metastatic patterns. This report serves as a powerful warning of the consequences of abandoning evidence-based adjuvant therapy in high-risk breast cancer.

## 1 Introduction

Breast cancer is the most common malignancy among women worldwide. The HER2-positive subtype, accounting for approximately 15-20% of all breast cancers, is characterized by aggressive biological behavior, including high proliferation rates, early recurrence, and a predilection for central nervous system (CNS) metastasis (1). The introduction of anti-HER2 targeted therapies–trastuzumab, pertuzumab, tyrosine kinase inhibitors such as lapatinib and pyrotinib, and the antibody-drug conjugate trastuzumab deruxtecan (T-DXd)–has dramatically altered the prognosis of this once-deadly subtype. T-DXd has shown promising activity in both early and metastatic settings, including the DESTINY-Breast06 and DESTINY-Breast11 trials (2). For patients with early-stage (stage I-II) HER2-positive breast cancer, adjuvant trastuzumab has improved 10-year survival rates to over 80% (3). However, for stage IIIA disease with multiple positive nodes, outcomes remain less favorable, with 10-year survival rates of approximately 50-60% even with optimal therapy. Our patient had stage IIIA disease (7/12 positive nodes) and declined all adjuvant treatment.

Despite these advances, some patients refuse standard adjuvant therapy due to personal beliefs, fear of toxicity, or preference for alternative medicine. Traditional Chinese medicine (TCM) is widely used in China as a complementary or alternative approach for cancer management. While TCM may offer symptomatic relief and immunomodulatory effects, its role as a sole systemic therapy for aggressive malignancies remains unproven (4).

Thyroid metastasis from breast cancer is exceptionally rare. While autopsy series report an incidence of 1-2% (5), recent clinical studies suggest an incidence of 7.8% to 13% (6). Breast cancer is actually one of the more common primaries to metastasize to the thyroid, but the overall rarity makes each case worthy of documentation. Brain metastasis in HER2-positive breast cancer, however, is a well-recognized complication, occurring in up to 30-50% of patients with advanced disease, particularly in those who do not receive agents capable of crossing the blood-brain barrier (7).

This case report details the clinical trajectory of a patient with high-risk HER2-positive breast cancer who declined all standard adjuvant therapy in favor of sole Chinese herbal medicine, subsequently developing metastases to the brain and thyroid. This case vividly illustrates the natural history of untreated HER2-positive disease and underscores the necessity of evidence-based adjuvant treatment.

## 2 Case presentation

### 2.1 Clinical presentation and history

A 63-year-old postmenopausal woman presented to our breast disease center in March 2026 with a chief complaint of progressively enlarging left neck lymphadenopathy for three years. She had a significant oncologic history: in March 2019, at age 55, she underwent a modified radical mastectomy of the left breast for invasive ductal carcinoma.

Her past medical history was otherwise unremarkable. There was no history of hypertension, diabetes, or other chronic illnesses. Family history was non-contributory for breast, ovarian, or other malignancies.

Critical oncologic history: Following her 2019 surgery, the patient was recommended to receive adjuvant chemotherapy (anthracycline plus taxane-based regimen), radiotherapy, endocrine therapy (given her ER-positive status), and anti-HER2 targeted therapy (trastuzumab). However, she declined all standard adjuvant treatments. Instead, she elected to pursue sole Chinese herbal medicine, the specific formulation of which was not documented in her medical records. She continued this regimen for approximately seven years until her current presentation.

### 2.2 Diagnostic workup and pathological confirmation (2019)

Initial diagnosis was established via core needle biopsy of the left breast mass in March 2019. Histopathological analysis revealed invasive ductal carcinoma, WHO grade II, with an extensive intraductal component (approximately 30%). Tumor size was 2.0 × 1.5 × 1.0 cm.

Immunohistochemistry (IHC) of the primary tumor showed positive ER (80-90%, moderate to strong intensity), negative PR, HER2 3+ (strong complete membranous staining), a Ki-67 proliferation index of 15%, PHH3–3 per 2mm², positive E-cadherin, membranous positive P120, negative P63, and negative CK5/6.

Fluorescence *in situ* hybridization (FISH) confirmed HER2 gene amplification with a HER2/CEP17 ratio of 5.60 (average HER2 signals/nucleus: 14.1; average CEP17 signals/nucleus: 2.52).

Pathological staging after modified radical mastectomy (March 20, 2019) revealed a pT1c (2.0 cm) primary tumor, pN2a status with 7 out of 12 axillary lymph nodes positive for metastasis, all surgical margins negative (skin, nipple, base), and no definite lymphovascular invasion identified by CD34, CD31, and D2–40 staining.

Following the pathological diagnosis, the patient was recommended to receive adjuvant chemotherapy, radiotherapy, endocrine therapy, and anti-HER2 targeted therapy (trastuzumab). However, she declined all standard adjuvant treatments and instead chose to pursue sole Chinese herbal medicine.

Histopathological images of the primary tumor are not shown due to fading of the original slides from 2019.

### 2.3 Current presentation (2026) and imaging findings

At presentation in March 2026, the patient reported no specific neurological symptoms, although a detailed review of systems was not documented. Physical examination revealed absence of the left breast and palpable, firm, poorly mobile left cervical lymphadenopathy. An ECOG performance status was not formally documented; based on the clinical description (ambulatory, able to perform light activities, NRS pain score 0), her status was estimated as 1.

No whole-body PET/CT was performed because the patient initially presented with localized neck symptoms and underwent targeted ultrasound evaluation. Thyroid ultrasonography was chosen as the initial imaging modality due to the presence of palpable cervical lymphadenopathy and the need to assess both thyroid and nodal status simultaneously. The ultrasound revealed suspicious thyroid nodules, prompting subsequent fine-needle aspiration. No additional distant metastatic foci (e.g., liver, lung, bone) were identified on the available imaging, but a comprehensive whole-body staging was not completed due to patient refusal of further investigations.

**Figure 1:**
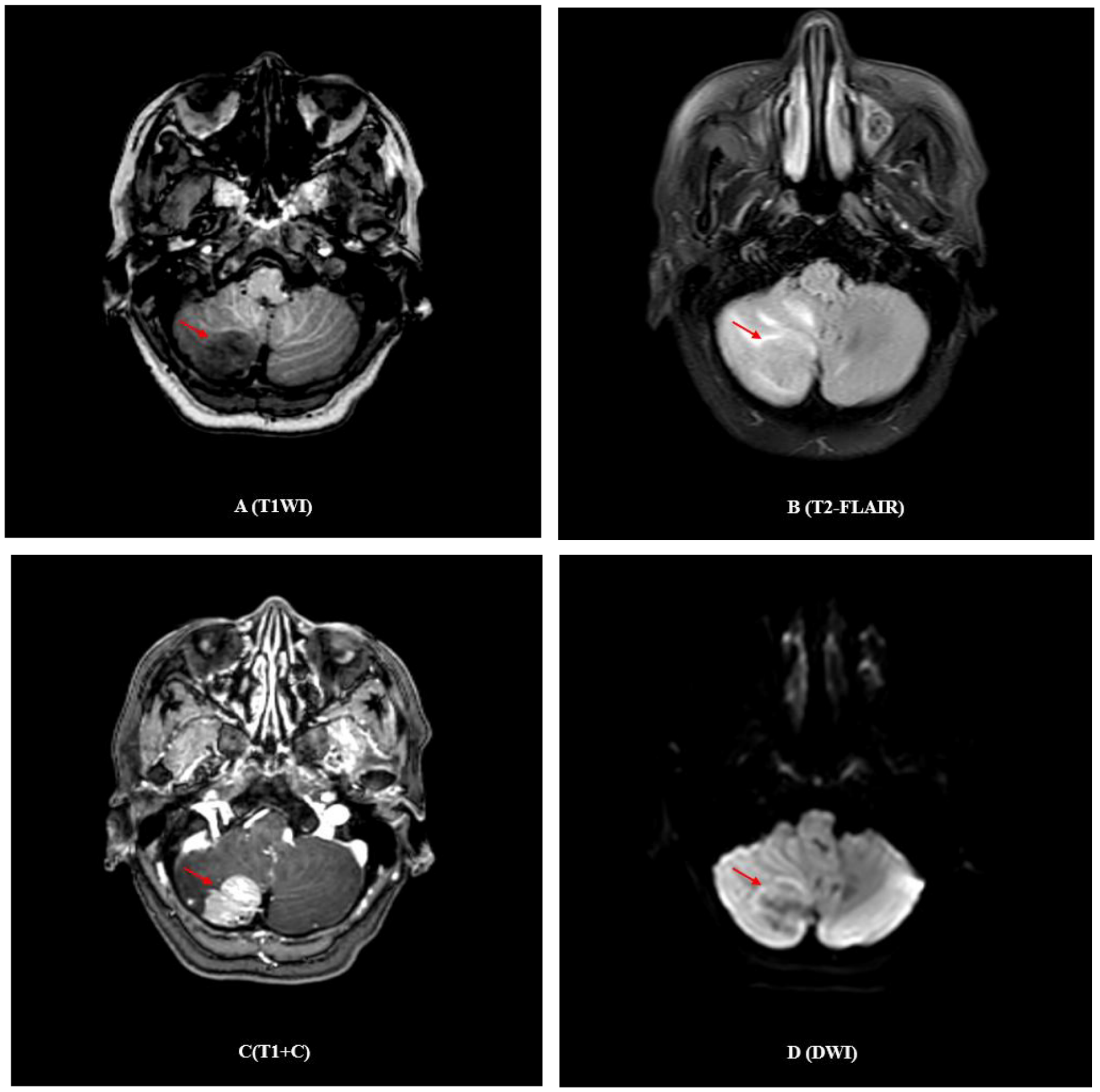
Brain MRI of the cerebellar metastasis. **(A)** T1-weighted imaging shows a hypointense lesion. **(B)** T2-FLAIR imaging demonstrates extensive peritumoral edema (hyperintense signal). **(C)** Post-contrast T1-weighted imaging reveals heterogeneous marked enhancement. **(D)** Diffusion-weighted imaging shows no significant diffusion restriction. The lesion measures 2.3 × 2.7 cm. The patient refused standard therapy and was followed with serial imaging.

Contrast-enhanced brain MRI (April 9, 2026) demonstrated a nodular lesion in the right cerebellar hemisphere measuring 2.3 × 2.7 cm. The lesion showed heterogeneous enhancement on T1-weighted imaging, high signal intensity on T2-weighted imaging with extensive peritumoral edema, and no significant diffusion restriction. The findings were highly suspicious for a metastatic tumor (**Figure 1**).

**Figure 2:**
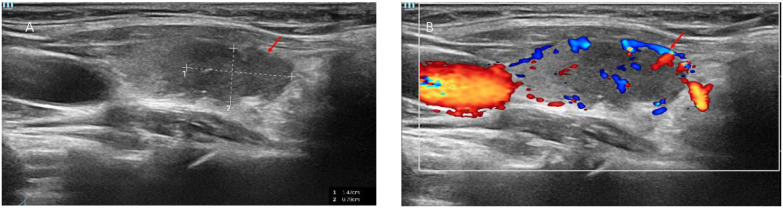
Thyroid ultrasound of the thyroid metastasis. **(A)** Gray-scale ultrasound shows a hypoechoic lesion in the right lobe, measuring 14.2 × 7.9 mm (calipers, lower right corner). **(B)** Color Doppler image demonstrates internal vascularity within the lesion.

Thyroid ultrasound revealed multiple suspicious nodules in the right thyroid lobe. An 8.1 × 6.1 × 6.6 mm hypoechoic nodule with ill-defined margins, irregular shape, and microcalcifications (TI-RADS category 4b) was identified. An adjacent hypoechoic area measuring 14.2 × 7.9 mm with scattered punctate echogenic foci and internal vascularity was also noted (**Figure 2**).

Cervical and axillary ultrasound demonstrated multiple abnormal lymph nodes in the left neck, left supraclavicular fossa, and left axilla. The largest measured 17.5 × 14.0 mm, with loss of fatty hilum, irregular margins, partial confluence, and internal vascularity.

Breast ultrasound of the right breast showed a 13.4 × 8.5 mm oval, well-circumscribed isoechoic mass (BI-RADS category 4A) and a 5.3 × 3.0 mm nodule (BI-RADS category 3). Biopsy of the BI-RADS 4A lesion was recommended but declined by the patient. Given the imaging features suggestive of a fibroadenoma, the lesion is being followed with serial ultrasound every 6 months. We acknowledge that a 2-10% malignancy risk remains. The left chest wall (post-mastectomy) showed no definite mass.

### 2.4 Pathological confirmation of metastatic disease (2026)

**Figure 3:**
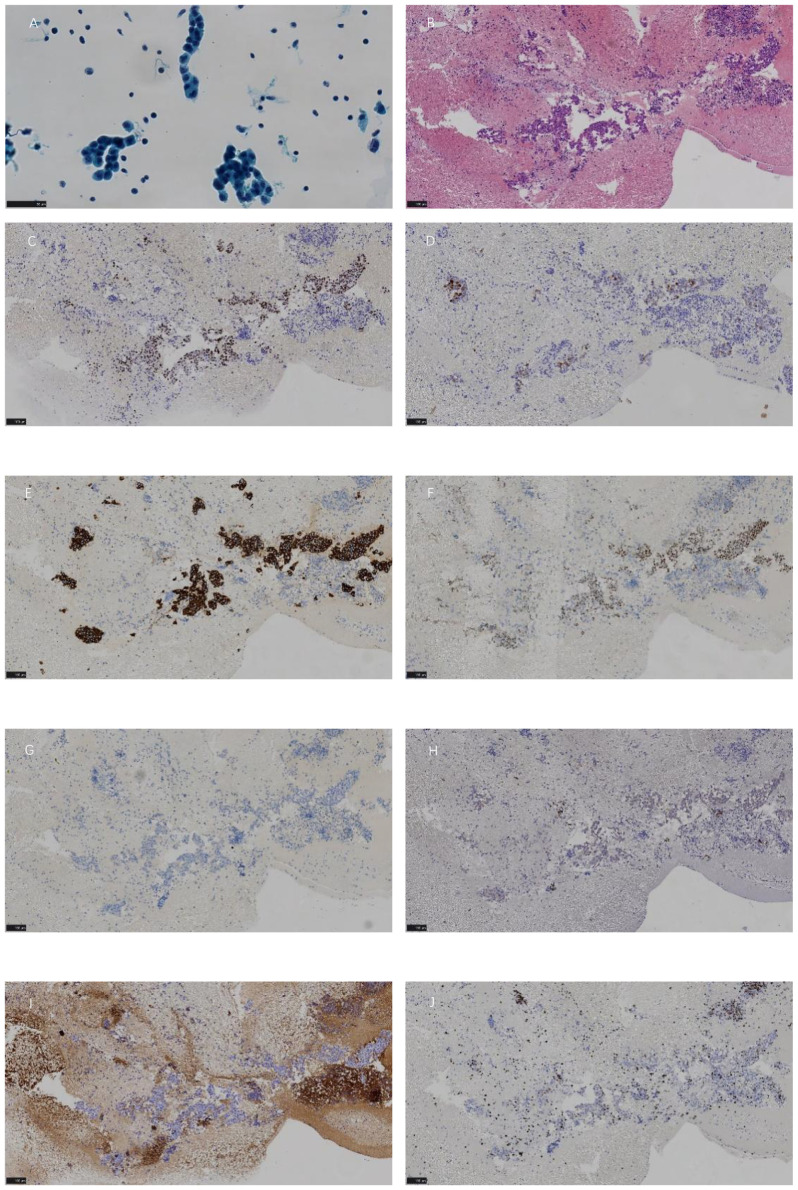
Cytology, cell block, and immunohistochemistry of the thyroid metastasis. **(A)** Liquid-based cytology shows atypical cells with high nuclear-to-cytoplasmic ratios and hyperchromasia (Papanicolaou stain, ×40). **(B)** Cell block preparation demonstrates clusters of malignant cells (H&E, ×10). **(C)** Tumor cells show diffuse nuclear positivity for GATA3 (×10). **(D)** GCDFP-15 is positive (×10). **(E)** HER2 shows strong complete membranous staining (3+) (×10). **(F)** ER is positive (80%, moderate to strong intensity) (×10). **(G)** PR is negative (×10). **(H)** TTF-1 is negative (×10). **(I)** Thyroglobulin (TG) is negative (×10). **(J)** Ki-67 proliferation index is approximately 15% (×10).

Thyroid fine-needle aspiration (FNA): Ultrasound-guided FNA of the right thyroid nodule was performed. Liquid-based cytology showed atypical cells with high nuclear-to-cytoplasmic ratios and hyperchromasia (**Figure 3A**). Cell block preparation revealed clusters of malignant cells (**Figure 3B**).

Immunohistochemistry (IHC) on the thyroid cell block: The profile was consistent with breast cancer origin: the tumor cells were positive for GATA3 (**Figure 3C**), GCDFP-15 (**Figure 3D**), HER2 (3+, strong complete membranous staining, **Figure 3E**), and ER (80%, moderate to strong intensity, **Figure 3F**); while negative for PR (**Figure 3G**), TTF-1 (**Figure 3H**), and thyroglobulin (TG, **Figure 3I**). The Ki-67 proliferation index was approximately 15% (**Figure 3J**).

Left axillary lymph node core biopsy: Histopathological examination revealed fibrous tissue infiltrated by malignant cells. Although IHC on the axillary node was not performed due to limited sample, the clinical and imaging context supported metastatic involvement.

Diagnostic conclusion: The combination of cytomorphology, cell block histology, and IHC profile (GATA3+/GCDFP-15+/HER2 3+/ER+/PR-/TTF-1-/TG-) definitively confirmed the diagnosis of metastatic breast carcinoma to the thyroid gland, with retention of the original HER2 and ER status.

### 2.5 Treatment and clinical course

Upon diagnosis of metastatic disease, the patient was referred to the medical oncology service. The following treatment recommendations were discussed with the patient:

- 2019 adjuvant regimen (at the time of initial diagnosis): The patient was recommended to receive adjuvant chemotherapy with an anthracycline plus taxane-based regimen (specifically dose-dense doxorubicin and cyclophosphamide followed by paclitaxel) combined with trastuzumab (AC-TH), followed by trastuzumab alone for one year. Endocrine therapy with tamoxifen or an aromatase inhibitor was also recommended given her ER-positive status (80-90%). The patient declined all.

- 2026 metastatic regimen: For metastatic disease with brain metastasis, the recommended first-line systemic therapy was dual HER2 blockade with trastuzumab and pertuzumab combined with a taxane (the CLEOPATRA regimen). The addition of a brain-penetrant agent such as tucatinib or pyrotinib is not a standard component of first-line therapy but has demonstrated intracranial activity in later lines; it was discussed as a potential option given the presence of brain metastasis. Endocrine therapy with letrozole or an aromatase inhibitor was also discussed due to retained ER positivity (80% in the thyroid metastasis). The patient declined all recommended therapies. Of note, the DESTINY-Breast09 trial is evaluating T-DXd as a potential new first-line standard.

- Local brain metastasis therapy: Stereotactic radiosurgery (SRS) was discussed as the primary local treatment option for the solitary 2.3 cm cerebellar lesion. Whole-brain radiotherapy was considered but not favored because of the risk of cognitive decline. The patient refused any form of radiation therapy.

As of the most recent follow-up (April 2026), the patient was alive but continued to decline standard systemic therapy.

At the last follow-up (April 2026), the patient reported intermittent mild headache and occasional unsteadiness when walking, but no nausea, vomiting, or visual changes. Her estimated ECOG performance status remained 1. She has been referred to the palliative care team for symptom management (specifically for headache and gait ataxia), and supportive care measures including dexamethasone as needed have been offered. The patient continues to refuse all conventional systemic therapy and radiation. A follow-up brain MRI with contrast is planned every 3 months to monitor the cerebellar metastasis, and neck ultrasound is planned every 6 months to assess thyroid and lymph node status. The clinical timeline is summarized in **Table 1**.

**Table 1:** Timeline of key clinical events and interventions.

Date/period	Category	Event and description	Key outcome/notes
March 2019	Diagnosis	Initial workup. Core needle biopsy of left breast mass.	Invasive ductal carcinoma confirmed.
March 2019	Biomarker	IHC and FISH testing.	ER 80-90%+, PR-, HER2 3+, FISH ratio 5.60, Ki-67 15%.
March 20, 2019	Surgery	Modified radical mastectomy with axillary lymph node dissection.	pT1cN2aM0 (Stage IIIA). 7/12 positive nodes. All margins negative.
March 2019	Treatment Decision	Patient refuses all standard adjuvant therapy (chemo, RT, endocrine, anti-HER2).	Patient opts for sole Chinese herbal medicine.
March 2019 – March 2026	Surveillance	No regular oncologic follow-up documented. Patient self-manages with Chinese herbal medicine.	Total follow-up on sole TCM therapy: ~7 years (disease-free interval approximately 4 years).
March 2026	Recurrence Presentation	Patient presents with left neck lymphadenopathy (present for 3 years, progressive).	Physical exam: palpable left cervical nodes.
March – April 2026	Diagnostic Workup	Brain MRI, thyroid ultrasound, lymph node ultrasound, FNA of thyroid, biopsy of axillary node.	Brain MRI: 2.3×2.7 cm right cerebellar lesion with edema (metastasis). Thyroid: TI-RADS 4b nodule; FNA suspicious for malignancy. Axillary node: carcinoma.
April 9, 2026	Diagnosis	Metastatic breast cancer confirmed (clinical and cytologic).	Brain metastasis, thyroid metastasis (IHC-confirmed), widespread lymph node metastasis.
April 2026	Treatment Planning	Patient referred to medical oncology for systemic therapy.	Anti-HER2 therapy + brain-penetrant agent recommended. Patient again declined standard therapy.

### 2.6 Patient perspective

According to clinical records, the patient was initially hopeful about her prognosis after surgery in 2019. She expressed a strong preference for natural or traditional therapies over what she perceived as toxic conventional treatments. She reported that her Chinese herbal medicine regimen was well-tolerated without significant side effects. However, she did not undergo regular surveillance imaging during the seven-year period, and the gradual enlargement of her neck lymph nodes over three years was not promptly evaluated.

Upon learning of the brain metastasis, the patient reportedly experienced significant emotional distress. Her perspective on the relative risks and benefits of conventional versus alternative therapy appeared to be evolving. This case highlights the importance of shared decision-making and the need for oncologists to address patient concerns about treatment toxicity while presenting evidence for the substantial survival benefits of standard adjuvant therapy in high-risk breast cancer.

## 3 Discussion

This case documents a sobering clinical course that underscores the aggressive natural history of untreated HER2-positive breast cancer. Despite being diagnosed at a potentially curable stage (pT1cN2aM0, Stage IIIA) with a moderate Ki-67 index (15%), the patient developed widespread metastatic disease—including a brain metastasis and an exceptionally rare thyroid metastasis—after seven years of sole Chinese herbal medicine therapy. This experience provides several important lessons for clinicians.

### 3.1 The consequences of refusing evidence-based adjuvant therapy

This patient’s primary tumor exhibited HER2 amplification and ER positivity, both drivers of aggressive behavior. For HER2-positive breast cancer, adjuvant trastuzumab reduces the relative risk of recurrence by approximately 50% and improves overall survival (3). For node-positive disease (7/12 positive nodes), the absolute benefit is even greater. Adjuvant endocrine therapy for 5–10 years would have further reduced her risk of recurrence (8). By declining all standard adjuvant therapy, the patient was effectively exposed to the full, unmodified natural history of her disease. The four-year disease-free interval and subsequent metastatic progression are consistent with known patterns of late recurrence in hormone receptor-positive/HER2-positive (Luminal B) tumors (9).

Beyond standard adjuvant therapy, emerging strategies may further improve outcomes. The PATINA trial investigates the addition of CDK4/6 inhibitors to endocrine therapy in patients with hormone receptor-positive/HER2-positive disease. Extended adjuvant neratinib (after trastuzumab) has been shown to reduce CNS recurrence in HER2-positive breast cancer. In the metastatic setting, trastuzumab deruxtecan (T-DXd) has demonstrated remarkable intracranial activity, with CNS response rates exceeding 50% in patients with active brain metastases (10). These options highlight the rapidly evolving landscape of HER2-directed therapy and underscore what this patient forewent by declining evidence-based treatment.

### 3.2 Thyroid metastasis: an extremely rare event

Breast cancer metastatic to the thyroid gland is a rare clinical entity. In large autopsy series, the incidence of thyroid metastasis from any primary ranges from 1.25% to 24%, with breast cancer being one of the more common sources (5). More recent clinical studies report an incidence of 7.8% to 13% for breast cancer metastatic to the thyroid (6). This discrepancy is explained by the fact that autopsy studies detect subclinical, asymptomatic metastases, whereas clinical studies are based on imaging or symptomatic cases. However, clinically evident thyroid metastases are much rarer; a current PubMed search identified approximately 135 reported cases in the English literature, and we have revised the text to state “fewer than 150 cases.” Similar cases of breast cancer metastasizing to unusual sites, including the thyroid and stomach, have been reported, underscoring the need for heightened diagnostic vigilance (11). A recent study identified age ≥60 years, menopausal status, and high Ki-67 index as risk factors for concurrent thyroid disease in breast cancer patients (12).

The clinical significance of identifying a thyroid metastasis is twofold. First, it must be distinguished from a primary thyroid malignancy, which would carry a different prognosis and treatment approach. The distinction requires immunohistochemistry: breast cancer metastases typically express GATA3, ER, and HER2 (if the primary was positive), while primary thyroid malignancies express TTF-1, PAX8, and thyroglobulin (13). In this case, immunohistochemistry on the thyroid FNA cell block (GATA3+, GCDFP-15+, HER2 3+, ER+, TTF-1-, TG-) definitively confirmed the metastatic origin and ruled out a primary thyroid malignancy.

Second, the presence of a thyroid metastasis indicates widespread hematogenous dissemination and is associated with a poor prognosis. In published case series, median survival after diagnosis of thyroid metastasis from breast cancer ranges from 12 to 36 months (5).

Although breast cancer is a common source of thyroid metastases, other malignancies such as renal cell carcinoma, lung cancer, and melanoma can also metastasize to the thyroid (14). The thyroid is generally considered an organ with relatively low metastatic involvement, and the presence of a thyroid nodule in a cancer patient should always raise suspicion for metastatic disease.

### 3.3 The role of traditional Chinese medicine in oncology

Traditional Chinese medicine is widely used in China and increasingly around the world as a complementary therapy for cancer patients. Several studies have investigated the potential immunomodulatory, anti-inflammatory, and direct anti-tumor effects of various TCM formulations (4).

However, it is critical to distinguish between complementary use (alongside standard therapy) and alternative use (in place of standard therapy). Numerous studies have shown that cancer patients who decline or delay standard therapy in favor of sole alternative medicine have significantly worse outcomes. A landmark study by Johnson et al. (2018) found that patients with breast cancer who refused conventional treatment and received alternative medicine alone had a 5-year mortality rate nearly six times higher than those receiving standard therapy (15).

In this case, the patient’s four-year disease-free interval and seven-year total follow-up after surgery-–despite her high-risk features-–is notable. It is not possible to determine whether this course reflects the natural history of hormone receptor-positive/HER2-positive breast cancer (which carries a well-documented risk of late recurrence) or any effect of the Chinese herbal medicine regimen. However, the eventual development of brain and thyroid metastases demonstrates that TCM alone was insufficient to eradicate micrometastatic disease. This case should not be interpreted as evidence for TCM efficacy, but rather as a cautionary tale about the limits of alternative medicine in aggressive malignancies.

### 3.4 Limitations

This case report has several limitations. First, the specific formulation of Chinese herbal medicine used by the patient was not documented, preventing any analysis of its potential biological effects. Second, immunohistochemistry on the axillary lymph node was not performed due to limited sample, although the thyroid metastasis was fully characterized. Third, a formal neurological examination (including cerebellar signs and signs of raised intracranial pressure) was not documented at the time of diagnosis of the brain metastasis. Fourth, no thyroid function tests, complete blood count, basic metabolic panel, or liver function tests were performed or documented at the time of metastasis diagnosis. Fifth, the patient has not yet initiated standard anti-HER2 therapy. Sixth, it remains unknown whether the patient would have remained recurrence-free had she received standard adjuvant therapy; even with optimal treatment, a subset of patients with stage IIIA HER2-positive disease still experience relapse. Finally, this is a single case and cannot provide generalizable evidence about the efficacy or lack thereof of any specific treatment.

### 3.5 Translational implications and future directions

This case raises important questions about tumor evolution under alternative treatment pressures. The IHC results from the thyroid metastasis allowed direct comparison with the primary tumor: ER status remained positive (80% vs. 80-90%), HER2 status remained 3+, and Ki-67 index remained 15%, indicating no significant molecular evolution after seven years without systemic therapy. However, the absence of axillary node IHC precluded further analysis. Future studies with paired primary and metastatic samples from patients who have undergone prolonged treatment-free intervals may help clarify the dynamics of clonal selection (16).

## 4 Conclusion

This case report starkly illustrates the aggressive natural history of untreated HER2-positive breast cancer. Despite a four-year disease-free interval (and seven years of total follow-up) during which the patient received sole Chinese herbal medicine, she ultimately developed widespread metastatic disease, including a brain metastasis and an exceptionally rare thyroid metastasis. This outcome underscores several critical messages for clinicians and patients:

First, evidence-based adjuvant therapy—including anti-HER2 targeted therapy, chemotherapy, and endocrine therapy—significantly improves outcomes in high-risk breast cancer and cannot be replaced by alternative medicine alone. Second, any new thyroid nodule in a breast cancer patient should be considered metastatic until proven otherwise. Third, brain metastasis in HER2-positive disease is a predictable complication in the absence of CNS-penetrant anti-HER2 agents.

It must be clearly emphasized that unregulated herbal treatments are not evidence-based, are often poorly standardized, and may delay or interfere with effective oncologic care. Patients with high-risk breast cancer should not use herbal medicine as a substitute for guideline-directed therapy.

The immunohistochemical characterization of the thyroid metastasis, which retained the original HER2 and ER profile, provides insight into the relative stability of this tumor’s molecular phenotype despite seven years without systemic therapy. We hope this case contributes to the literature on rare metastatic patterns and serves as a powerful reminder of the life-saving potential of modern adjuvant therapy.
